# Oncogenic Intra-p53 Family Member Interactions in Human Cancers

**DOI:** 10.3389/fonc.2016.00077

**Published:** 2016-03-31

**Authors:** Maria Ferraiuolo, Silvia Di Agostino, Giovanni Blandino, Sabrina Strano

**Affiliations:** ^1^Translational Oncogenomics Unit, Department of Molecular Medicine, Regina Elena National Cancer Institute, Rome, Italy; ^2^Molecular Chemoprevention Unit, Department of Molecular Medicine, Regina Elena National Cancer Institute, Rome, Italy

**Keywords:** p53 gene family members, gain of function, homology, isoforms, protein–protein interaction, target genes, apoptosis, differentiation

## Abstract

The p53 gene family members p53, p73, and p63 display several isoforms derived from the presence of internal promoters and alternative splicing events. They are structural homologs but hold peculiar functional properties. p53, p73, and p63 are tumor suppressor genes that promote differentiation, senescence, and apoptosis. p53, unlike p73 and p63, is frequently mutated in cancer often displaying oncogenic “gain of function” activities correlated with the induction of proliferation, invasion, chemoresistance, and genomic instability in cancer cells. These oncogenic functions are promoted either by the aberrant transcriptional cooperation of mutant p53 (mutp53) with transcription cofactors (e.g., NF-Y, E2F1, Vitamin D Receptor, Ets-1, NF-kB and YAP) or by the interaction with the p53 family members, p73 and p63, determining their functional inactivation. The instauration of these aberrant transcriptional networks leads to increased cell growth, low activation of DNA damage response pathways (DNA damage response and DNA double-strand breaks response), enhanced invasion, and high chemoresistance to different conventional chemotherapeutic treatments. Several studies have clearly shown that different cancers harboring mutant p53 proteins exhibit a poor prognosis when compared to those carrying wild-type p53 (wt-p53) protein. The interference of mutantp53/p73 and/or mutantp53/p63 interactions, thereby restoring p53, p73, and p63 tumor suppression functions, could be among the potential therapeutic strategies for the treatment of mutant p53 human cancers.

## Introduction

**Figure 1:**
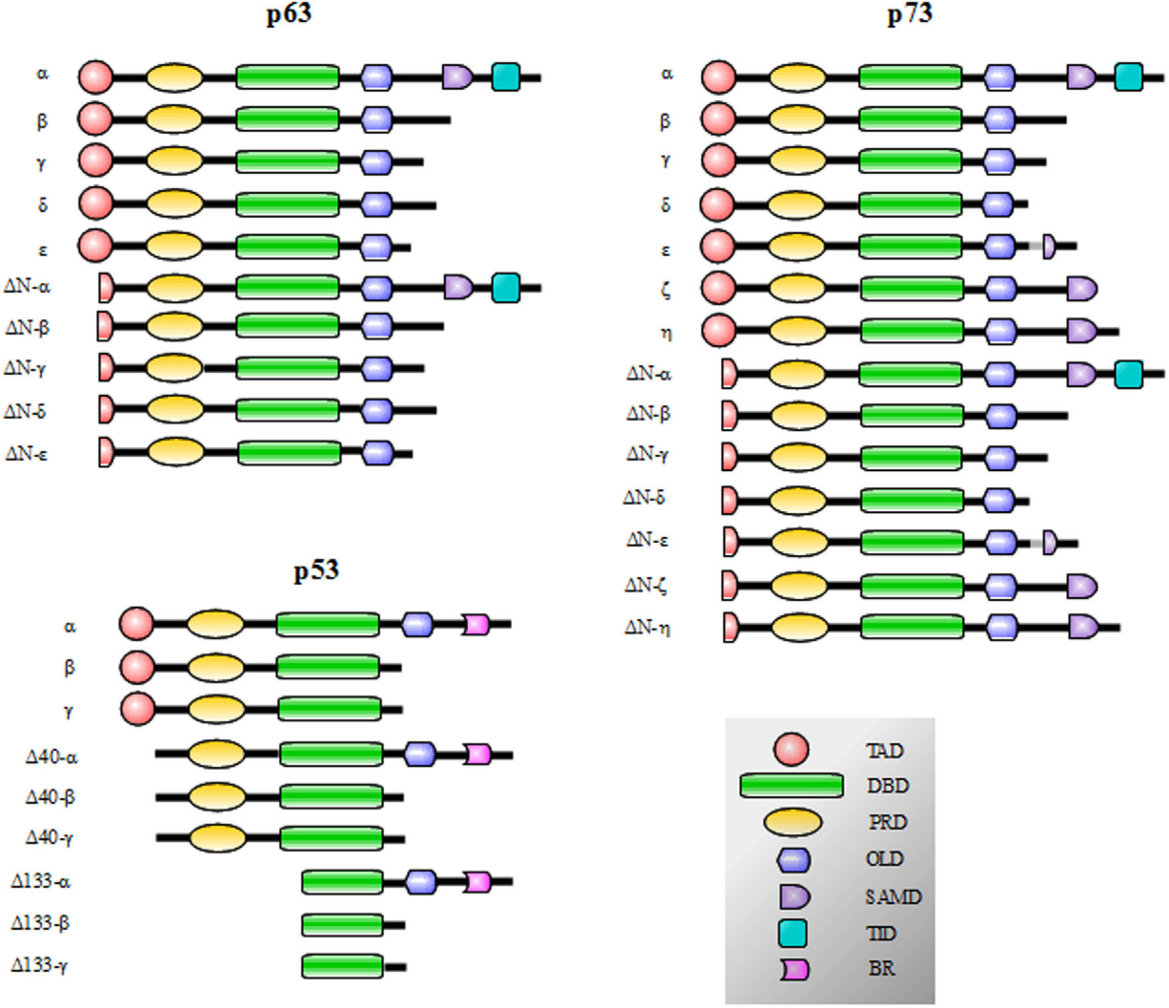
**p53, p73, and p63 isoforms obtained by the presence of internal promoters in the sequence (ΔN isoforms) and by alternative splicing**. The N-terminal transcription-activation domain (TAD) contains two subdomains (AD1 and AD2) and is the binding-site for regulators of gene transcription; in p63 and p73 proteins, the C-terminal transactivation inhibitory domain (TID) binds to the TAD preventing constitutive transcriptional activity. The Proline-rich sequence recognition domains (PRD) can recognize proline-rich motifs of interacting proteins and has been reported to be essential for the induction of apoptosis driven by p53, p73 and p63. The DNA-binding domain (DBD) is responsible for the binding to DNA consensus of target genes and the oligomerization domain (OD) enables monomers assembly in active oligomers. The sterile alpha motif domain (SAMD) of p63 and p73 is arranged in a small five-helix bundle and is involved in protein–protein interactions. The basic region (BR) in the C-terminal of p53 is involved in the control of the DNA binding affinity.

p53, p73, and p63 proteins belong to a family evolutionarily conserved in animals. They derive from an ancestral gene by duplication and consequent divergence of the original sequence. Functional and phylogenetic analyses reveal that the founding member was p63, followed by p73 and lastly p53 (1–4). In fact, at the sequence level, p63 and p73 display elevated homology to each other, more than to p53 (5–9). Generally, the protein structure consists of a central DNA-binding domain (DBD) (core domain) that binds to response elements of target genes (10–14). The N-terminal transcription-activation domain (TAD) is the binding-site for positive (e.g., p300/CBP and TAFII40/60) or negative regulators (e.g., MDM2 and MDMX) of gene transcription (15). The C-terminal oligomerization domain (OD) is subject to splicing and post-translational modifications, and it has been shown to influence DNA binding and transcriptional activity of the p53 family members (16) (Figure 1).

The human *TP53* gene (Chr.17p13.1) is about 20 kb, contains 11 exons and encodes for the tumor suppressor p53 protein known as “the guardian of the genome” (17–28). This protein was discovered in 1979 (29–31). p53 is not only able to act as a transcription factor but it is also involved in transcription-independent response, such as apoptosis (32). *TP53* is the most frequently mutated gene in human cancers (33–36). It is mostly affected by missense mutations often in the DBD of the protein (37–39). An increasing number of p53-mutated proteins can be distinct in either conformational mutants (when the mutations change the tridimensional structure of the protein) or in DNA-contact defective mutants (when the mutations affect the region designate for the binding to the DNA) (36, 40). These alterations lead to the inability of mutant p53 proteins to fully recognize the DNA consensus sequence for wt-p53 or alter the functional interaction with pro-apoptotic partners, such as WW domain-containing oxidoreductase WOX1 (WWOX). The WWOX/wt-p53 complex is demonstrated to induce apoptosis synergistically and WWOX is essential for p53 activation and apoptosis induction (41). Strikingly, some of the p53-mutated proteins acquire new oncogenic functions [gain of function (GOF)] that strongly contribute to increasing cell proliferation, invasion, angiogenesis, genomic instability and chemoresistance in human cancers. These functions are often promoted by the interaction with sequence-specific transcription factors and the consequent activation/repression of specific target genes diverse from those recruited by wt-p53 (15, 42–58).

The *TP73* gene (Chr.1p36.33) is about 65 kb and contains 14 exons. It was discovered in 1997 (59) and similar to its family members, plays an important role at different regulatory checkpoints of the cell cycle (60). However, p73 shows a peculiar function in neuronal differentiation, not shared with p53 (8, 61). p73 is rarely affected by mutation in cancer progression (62, 63) but its expression is deregulated in several human tumors, such as in hepatocellular carcinoma (64), neuroblastoma (65), lung cancer (62), prostate cancer (66) and colorectal carcinoma (67). p73 shares many p53 tumor suppression functions through the activation of the p53-target genes (p21^waf1^, Bax, PUMA, NOXA, IGF-BP3 and Cyclin G), resulting in the control of cell proliferation, differentiation, development and induction of apoptosis (68–79). Moreover, p73, like p53, can interact with the tumor suppressor WWOX and trigger apoptosis (80, 81). Cells exposure to DNA-damaging agents (e.g., γ-radiation and cisplatin) induces p73 protein activation and accumulation with consequent induction of DNA damage response (DDR), growth arrest and apoptosis (76, 82–88).

The *TP63* gene (Chr.3q27–29) is approximately 65 kb and contains 15 exons (89, 90). Like p73, p63 can activate many p53-target genes in response to oncogenic stress or DNA damage (Bax, 14-3-3σ, p53AIP1, IGF-BP3, p21^waf1^ and cyclin G), it controls cell proliferation, apoptosis, differentiation and development, and shows a tissue-specific localization (79, 91–94). p63 knockout mice exhibit a lethal phenotype soon after birth. They suffer from epithelial abnormalities, concerning skin, glands, teeth and hair follicles (95). Mutations of p63 are extremely rare in malignancies in contrast to p53 mutations. However, alterations in p63 expression patterns play an important role in tumorigenesis (96–98).

The full-length forms of p73 and p63 can also bind to YAP protein in response to DNA-damaging agents and activate pro-apoptotic target genes, such as Bax and p53AIP1 (78, 87, 99–101). Thus, both p73 and p63 can promote p53-independent apoptosis (102).

## Protein Structure and Respective Isoforms of the p53 Family Gene Members

p53 family gene members show a high degree of similarity in the exon/intron organization and share a similar modular protein structure previously described (Figure 1). p73 and p63 proteins display 22–29% of homology in the TAD domain, 63% in the DBD, and 42% in the OD of p53. Furthermore, critical residues in the DBD, involved in the folding and binding to target DNA sequences, are strictly conserved (22, 103–106). Moreover, p73 and p63 share a sterile alpha motif domain (SAMD) and a transactivation inhibitory domain (TID); p53, p63, and p73 contain also a proline-rich sequence recognition domain (PRD). p53 also shows an additional basic region (BR) in the C-terminal tail (16, 107, 108) (Figure 1).

p53 protein displays nine isoforms obtained by the presence of cryptic internal promoters and by alternative splicing. The result is the presence of potentially transcript inert isoforms N-terminal deleted (ΔN) and with various C-terminal tails (108) (Figure 1). The same mechanisms occur for p73 and p63: p73 displays 14 isoforms and p63 exhibits 10 isoforms (59, 90, 109–117) (Figure 1). The N-terminal truncated isoforms ΔNp73 and ΔNp63 are highly expressed in the development and display an oncogenic dominant-negative function to p73 and p63 full-length (TAp73 and TAp63, respectively) and wt-p53 (39, 114, 118–124).

## mutp53, p73, and p63 Protein Interactions in Cancer

It was observed that human tumor-derived p53 mutants could bind p73α interfering with its transcriptional activity and impeding apoptosis induction (125, 126). Strano et al. (127) demonstrate that p53 mutants (p53R175H and p53D281G) associate with four p73 isoforms *in vitro* and *in vivo* (p73α, p73β, p73γ and p73δ). The interactions occur also in physiological conditions in breast cancer cell lines (T47D and SKBR3) and require the DBD of mutp53 and the DBD and OD domains of p73 isoforms. Marin et al. (126) show that the interaction between mutp53 and p73α or p73β is also governed by a polymorphism at the codon 72 of the p53 mutant proteins (e.g., mutp53R175H and mutp53V143A) that encode for Arginine (R) or Proline (P). Particularly, p53 mutants with R72 polymorphism favor binding to p73 more than the P72 polymorphism determining poor response to therapy and poor prognosis in patients (128, 129). Thus, either the type of p53 mutation and 72R/P polymorphism determine mutp53/p73 interaction (126). The mutp53/p63 interaction, *in vitro* and in tumor cells, is also reported (126). Gaiddon et al. (130) demonstrate that p73α, p73β, p73γ, and p73δ can interact with overexpressed or endogenous p53 mutants (R175H, H179R, Y220C, R248W and R273H) and demonstrate that p53 mutants (R175H, Y220C and R248W) can bind to p63α and p63γ. Low-affinity interactions are observed between mutp53R175H and ΔNp63α or ΔNp63γ. Moreover, they observe that the interaction between p73α and p63α or ΔNp63α is more efficient if p73α is mutated (R292H). Gaiddon et al. (130) confirm that p53 mutants require the DBD domain for the interaction with p73 or p63. Moreover, p53 mutants deleted in several regions, resulting in conformational changes of the DBD, are still able to bind p73 and p63. Thus, also the wild-type DBD of mutp53 can interact with p73 or p63 if it is in a mutant conformation (130, 131). It is also demonstrated that the heat shock protein HSC70 binds those p53 mutants that interact with p73 but not wt-p53 (130, 132), thus, other determinants could affect p53/p73 interaction (130). Strano et al. (46) demonstrate that, under physiological conditions, mutp53 interacts with p63α and p63γ in T47D and HaCat cells and in H1299 cells overexpressing mutp53R273H or mutp53R248W. They observe direct interactions mediated by the DBDs of mutp53 and p63. Mutp53D281G displays a GOF activity, it slightly binds to p73 but does not interact with p63 (46, 126, 127, 133). Moreover, mutp53D281G mutated in the TAD loses its GOF, suggesting that the TAD exerts an important oncogenic role in the GOF of this p53 mutant (15, 134, 135). Santini et al. (136) provide biochemical evidence on the interaction between mutp53R175H and p73. They use atomic force spectroscopy (AFS) (137, 138) and surface plasmons resonance (SPR) (139, 140), identifying a high interaction force and a dissociation equilibrium constant typical for specific bounds. They do not observe any interactions between wt-p53 and p73 (136), confirming the lack of *in vivo* evidence for the formation of wt-p53/p73 protein complex. Weissmueller et al. (141) confirm that mutant p53 is able to bind p73 and this interaction results in the reduction of p73/NF-Y inhibitory complex in pancreatic ductal adenocarcinoma. This complex displays a tumor suppressor function repressing the oncogenic platelet-derived growth factor receptor b (PDGFRb) that promotes cell invasion and metastasis. Therefore, indirectly, mutant p53 promotes PDGFRb expression disassembling the inhibitory p73/NF-Y complex. Liu et al. (142) show that TopB1 protein promotes mutp53/NF-Y and mutp53/p73/p63 complex formation, inducing chemoresistance and proliferation in cancer cells. Wang and Fersht (143) describe the aggregation kinetics of mutant p53 that co-aggregate in tetramers by trapping also wt-p53, p73, and p63 proteins in the complex. It is worth to mention the role of MDM2 in the mutp53, p63, and p73 interplay: a recent work shows that MDM2, a negative regulator of wt-p53, competes with p63 for binding to mutp53R175H and in this way p63 activity is restored; but, on the other hand, MDM2 forms a trimeric complex with p73 and mutp53R273H strongly inhibiting p73 function (144). All these are clear examples of how different mutations in p53 protein could determine distinct protein–protein interactions and cell responses.

## Functional Implication of mutp53/p73/p63 Protein Interactions in Cancer

Knockout mice for p53^−/−^, p73^−/−^ and p63^−/−^ highlight the major physiological roles of these proteins (96), suggesting pivotal functions in the development of nervous and immune systems (mediated by p73), in skin and limb development (mediated by p63) and in tumor suppression (mediated primarily by p53) (111, 145–148).

The role of p73 and p63 in tumorigenesis (p53-dependent or independent) is controversial. In many tumors, these proteins are downregulated, in others they are overexpressed or their genes are amplified. This apparent incongruence is mainly due to the different isoforms, tissue-specific localization and functions exerted by these proteins (121, 130, 149, 150). The decisive effect depends on the ratio TA/ΔN of p73 and p63 isoforms, p73/p63 interactions and p73/p63 binding to the promoters of p53-target genes. (151–155).

**Figure 2:**
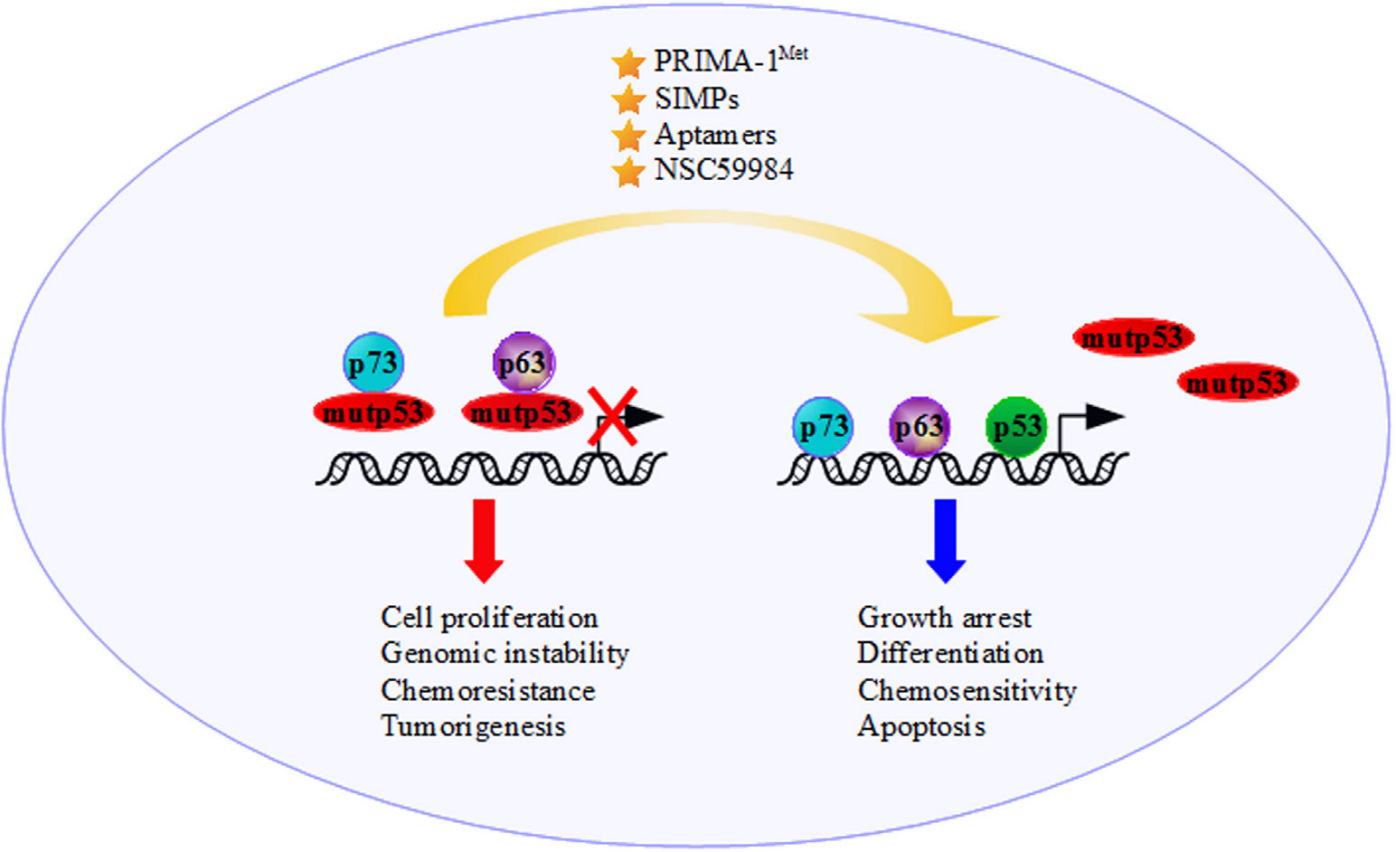
**The presence of mutant p53 moves the tumor suppressor functions of p73 and p63 to an oncogenic outcome through binding and inactivating p73 and p63 transcriptional activity**. The development of new anticancer strategies, such as increasing p73/p63 activities and employing molecules that interfere with mutantp53/p73 and/or mutantp53/p63 interactions [e.g., the molecule p53 reactivation and induction of massive apoptosis (PRIMA-1^Met^), short interfering mutp53 peptides (SIMPs), NSC59984 and Aptamers], could restore the p53 family’s tumor suppressor functions.

The status of p53 in cancer cells is a determining factor in the response to anticancer treatments (156–159). Some of the GOF activities mediated by mutp53 are related to the interaction, and consequent inactivation, of p73 and p63 (Figure 2). For example, the increase in chemoresistance to etoposide or cisplatin might involve the mutp53-dependent inactivation of p73-induced cell death (127, 128, 160–162). Importantly, in the presence of mutant p53 is observed a marked reduction of the transcriptional activity of p73α, p73β, p73γ and p73δ on the p21^waf1^ promoter (127). Gaiddon et al. (130) show that the interaction between p73 and p53 mutants (R175H, Y220C and R283H) reduces p73 transactivation of the p21^waf1^ promoter, highlighting the correlation between the capability of p53 mutants to interact with p73 and inhibit its transcriptional activity. Similar results are obtained for those p53 mutants that bind to p63α and p63γ reducing p63 activation of the p21^waf1^ promoter (130). When mutp53 binds to the OD domain of p73, it causes the functional inhibition of p73, impairing the interactions with other modulators (162). Furthermore, when mutp53 binds to the DBD of p73, it provokes a physical sequester of p73 from the consensus sequences on the target genes (127, 163, 164). Similarly, mutp53/p63 interaction results in p63 impairment of the transcriptional activation of its target genes (Bax, p21^waf1^, Cyclin G, 14-3-3σ and p53AIP1) (46). The formation of mutp53/p63 complex is also directly related to promoting cell invasion and metastasis in several cancer cell lines through mutp53-dependent inactivation of TAp63 tumor suppression functions (165–167).

In the past, many studies were dedicated to restoring the wild-type activity of the mutant p53 proteins (168). A great number of small molecules, aiming to restore and stabilize the original DBD conformation of p53, have been developed, such as p53 reactivation and induction of massive apoptosis (PRIMA-1) and maleimide-derived molecule MIRA-1. These compounds showed great promise when tested in cancer cell lines, demonstrating the induction of apoptotic processes (169–172). Unfortunately, the application of these molecules in the clinical practice is far off because the increased activity of p53 subjects non-cancerous cells to apoptosis induction. Further research is needed to minimize the level of cell toxicity (173). Another approach, proposed by Di Agostino et al. (174), refers to the disassembling of the mutp53/p73 complex using short interfering mutp53 peptides (SIMPs) (10–15 residues) that compete specifically with p73 for the binding of mutp53 to the DBD. This results in releasing p73 from the complex, activating apoptosis and rescuing cells from chemosensitivity. Notably, SIMPs have no cytotoxic effects on cells carrying wt-p53 proteins (174).

PRIMA-1^MET^ (APR-246, developed by APREA), a compound very similar to PRIMA-1 but much more active at low dosage, is discovered to restore mutant p53 (R273H and R175H) activity *in vitro* and *in vivo* (170, 175). Interestingly, PRIMA-1^MET^ not only restores the pro-apoptotic function of p53 but is also involved in activating downstream target genes of the p53 family (176, 177). PRIMA-1^MET^ alone and in combination with chemotherapeutic drugs are effective to induce apoptosis *in vivo* (178, 179). PRIMA-1^MET^ has also successfully completed a Phase I clinical trial, showing a promising efficacy (https://www.clinicaltrials.gov/ct2/show/NCT00900614). This molecule seems to lead to the formation of covalent adducts on mutant p53R175H and p53R273H proteins, but the exact mechanism of action has yet to be fully elucidated (180). Moreover, recently it is discovered that a small molecule, NSC59984, can restores wt-p53 signaling via p73 activation and induces p73-dependent cell death in colorectal cancer cells, without evident toxicity toward normal cells (181).

## Conclusions

Flores et al. (164) demonstrated that wt-p53, p73 and p63 are recruited onto regulatory regions of the p53-target genes inducing growth arrest, differentiation, senescence and apoptosis (Figure 2). Despite this, the DBD of mutp53, previously regarded as “dead domain” since it could not bind to the wt-p53 binding site of its target genes, acquires a new protein–protein interaction function sequestering and inactivating tumor suppression proteins, including the family members p73 and p63. This mechanism contributes to the GOF activity of mutp53 (9).

## Future Perspectives

The precise tackling of GOF activity of mutant p53 might lead to the discovery of drugs with broad anticancer effects. As far as our knowledge on the molecular mechanisms governing mutant p53 oncogenic activities advances, we have learned that mutant p53 proteins are not a single entity but a protein family with high intrinsic complexities. Since mutant p53 is a partner of oncogenic multi-protein complexes, one way to severely defeat its pro-tumorigenic activity may reside in the specific targeting of its key cooperative partners. Along this line of evidence, agents that increase p73 and/or p63 activity promoting chemosensitivity could represent a promising strategy to treat tumors harboring mutant p53 proteins (122, 182, 183) (Figure 2). It could be useful to develop and validate reagents that interfere with mutp53/p73 and mutp53/p63 interactions restoring p53, p73, and p63 tumor suppression functions. NSC59984, PRIMA-1^MET^, SIMPs and peptide aptamers, which bind specifically to mutant p53, could be a potent strategy in cancer therapy for these tumors (174, 181, 184) (Figure 2). Undoubtedly, greater knowledge must be acquired regarding the determinants of these oncogenic multi-protein complexes in order to design and pave novel therapeutic strategies to successfully treat mutant p53 human tumors.

## Author Contributions

The authors, MF and SDA, provided the structure and the writing of the article, which was integrated and supervised by SS and GB.

## Conflict of Interest Statement

The authors declare that the research was conducted in the absence of any commercial or financial relationships that could be construed as a potential conflict of interest.
